# Clinical outcomes according to the average daily dose of sacubitril/valsartan: a nationwide longitudinal cohort study

**DOI:** 10.1007/s00392-025-02602-x

**Published:** 2025-03-18

**Authors:** Jaehyun Lim, Hyun-Jung Lee, Soongu Kwak, Bongseong Kim, Kyung-Do Han, Heesun Lee, Jun-Bean Park, Yong-Jin Kim, Hyung-Kwan Kim

**Affiliations:** 1 Department of Internal Medicine, Seoul National University Hospital, 101 Daehak-Ro, Jongno-Gu, Seoul, 03080 Republic of Korea; 2 Department of Internal Medicine, Seoul National University College of Medicine, Seoul, Republic of Korea; 3 Division of Cardiology, Severance Hospital, Yonsei University College of Medicine, Seoul, Republic of Korea; 4 Department of Statistics and Actuarial Science, Soongsil University, Seoul, Republic of Korea; 5 Division of Cardiology, Department of Internal Medicine, Seoul National University Hospital Healthcare System Gangnam Centre, Seoul, Republic of Korea; 6 Section of Cardiovascular Imaging, Division of Cardiology, Cardiovascular Center, Seoul National University Hospital, Seoul, Republic of Korea

**Keywords:** Angiotensin receptor–neprilysin inhibitor, Submaximal dose, Heart failure, All-cause mortality, Heart failure management

## Abstract

**Aims:**

A minority of patients with heart failure (HF) are prescribed the maximal dose of the angiotensin receptor–neprilysin inhibitor sacubitril/valsartan. We investigated the effectiveness of submaximal doses of sacubitril/valsartan in a real-world cohort.

**Methods and results:**

Patients with HF with reduced ejection fraction prescribed sacubitril/valsartan for ≥ 180 days between 2016 and 2020 were included from a nationwide database, and categorized into tertiles based on the average daily sacubitril/valsartan dosage. Baseline characteristics were balanced using inverse probability of treatment weighting with propensity scores. The primary outcome was a composite of HF hospitalization and all-cause mortality. The study included 3,953 patients (age 62.6 ± 12.4 years, 73.0% men). Patients on lower sacubitril/valsartan doses were older, more likely to be women, and had more comorbidities, with lower blood pressure, reduced kidney function, and lower body mass index; however, baseline characteristics were well balanced across the groups after weighting. During a mean follow-up of 2.0 ± 0.7 years, there were 808 events (20.4%). The risk of the primary outcome in the middle (HR 0.93, 95% CI 0.78–1.10) and the highest dosage tertiles (HR 0.88, 95% CI 0.74–1.06) did not significantly differ compared with the lowest dosage tertile (*p*-value = 0.384). Regarding individual outcomes, there was no significant difference in HF hospitalization; however, there was a trend toward lower mortality with higher sacubitril/valsartan dose (*p*-value = 0.047).

**Conclusions:**

No significant difference was observed in the composite risk of HF hospitalization and all-cause mortality across different sacubitril/valsartan dosage groups. This suggests that the benefits of sacubitril/valsartan treatment may not necessarily be dose-dependent.

**Graphical Abstract:**

Clinical characteristics of patients according to sacubitril/valsartan dosage tertiles and their clinical outcomes. Patients in the lowest dosage tertile were more likely to be women and had unfavourable clinical characteristics. However, after inverse probability of treatment weighting, the Kaplan–Meier curves revealed no significant differences with regard to heart failure hospitalization, all-cause mortality, and the combination of these.

**Figure:**
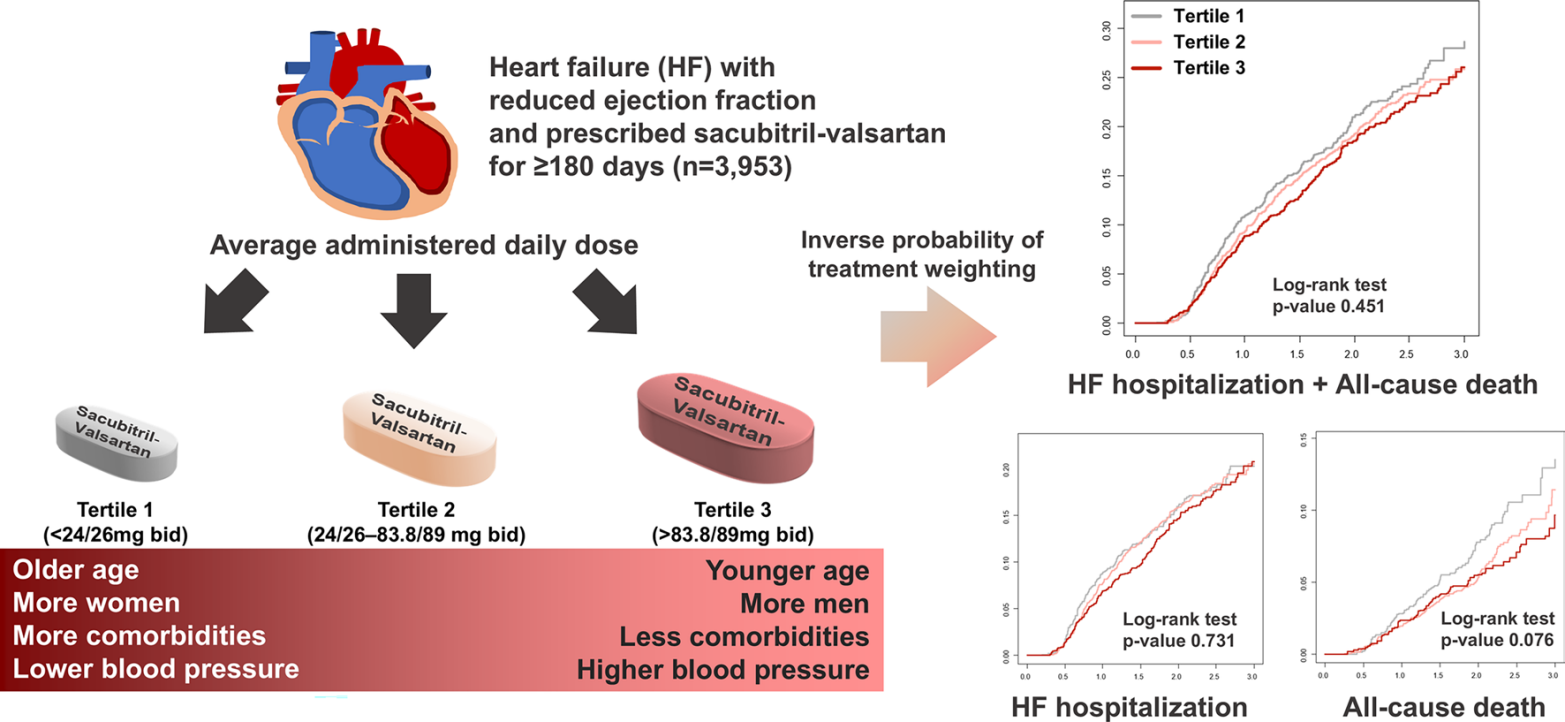

**Supplementary Information:**

The online version contains supplementary material available at 10.1007/s00392-025-02602-x.

## Introduction

The management of heart failure (HF), particularly HF with reduced ejection fraction (HFrEF), has entered a new era with the advent of a new drug class, angiotensin receptor–neprilysin inhibitors, comprising sacubitril/valsartan. Since the landmark PARADIGM-HF (Prospective Comparison of Angiotensin Receptor Neprilysin Inhibitor with Angiotensin-Converting Enzyme Inhibitor[ACEi] to Determine Impact on Global Mortality in HF) trial, sacubitril/valsartan has become a Class I recommendation in the treatment guidelines for HFrEF [1–4]. However, the external validity of the PARADIGM-HF warrants cautious interpretation, as it exclusively included patients who could tolerate a high dose of 97/103 mg sacubitril/valsartan twice daily (400 mg/day) during a run-in period before randomization. Indeed, the feasibility of such high doses has frequently been questioned, and several real-world studies have reported that only 15.8–27.2% of HFrEF patients taking sacubitril/valsartan were prescribed the target dose of 97/103 mg twice daily (totaling 400 mg/day) [5–8]. Reaching target doses of all HF medications is often unfeasible, and less than 1% of patients in a large HFrEF registry were concomitantly treated with target doses of HF medications [9]. Simultaneously, the strategy of using maximal doses of all HFrEF medications is also under scrutiny, as certain populations have shown similar benefits with submaximal doses [10–12].

A recent study reported that patients using lower doses of sacubitril/valsartan had similar improvements in the serum levels of biomarkers, symptoms, and cardiac reverse remodelling compared with those using higher doses [13]. Notwithstanding, no hard outcomes have been reported. With this background, we hypothesized that cardiovascular outcomes among patients receiving lower doses of sacubitril/valsartan would not differ significantly from those receiving higher doses. To confirm this hypothesis, we utilized a real-world population-based cohort encompassing the entire HFrEF population in Korea.

## Methods

### Study participants and design

The present study utilized the Korean National Health Insurance Service (NHIS) database, which encompasses comprehensive claims data on overall healthcare utilizations in Korea: these include hospital visits, sociodemographic data, diagnoses, conducted procedures, prescriptions, and mortality data. In addition, the NHIS Health Screening Program database, which is linked to the NHIS database, was leveraged to obtain crucial health-related data pertinent to the study outcomes, such as body mass index (BMI), estimated glomerular filtration rate (eGFR), and blood pressure. Korean citizens are mandated to undergo health screening including anthropometric measurements, questionnaires on health behaviors, and blood tests every 1 or 2 years. This study adhered to the ethical standards of the Declaration of Helsinki and was approved by the Institutional Review Board of Seoul National University Hospital (2401–034-1500). Informed consent was waived owing to the retrospective and anonymized nature of the NHIS data.

**Fig. 1:**
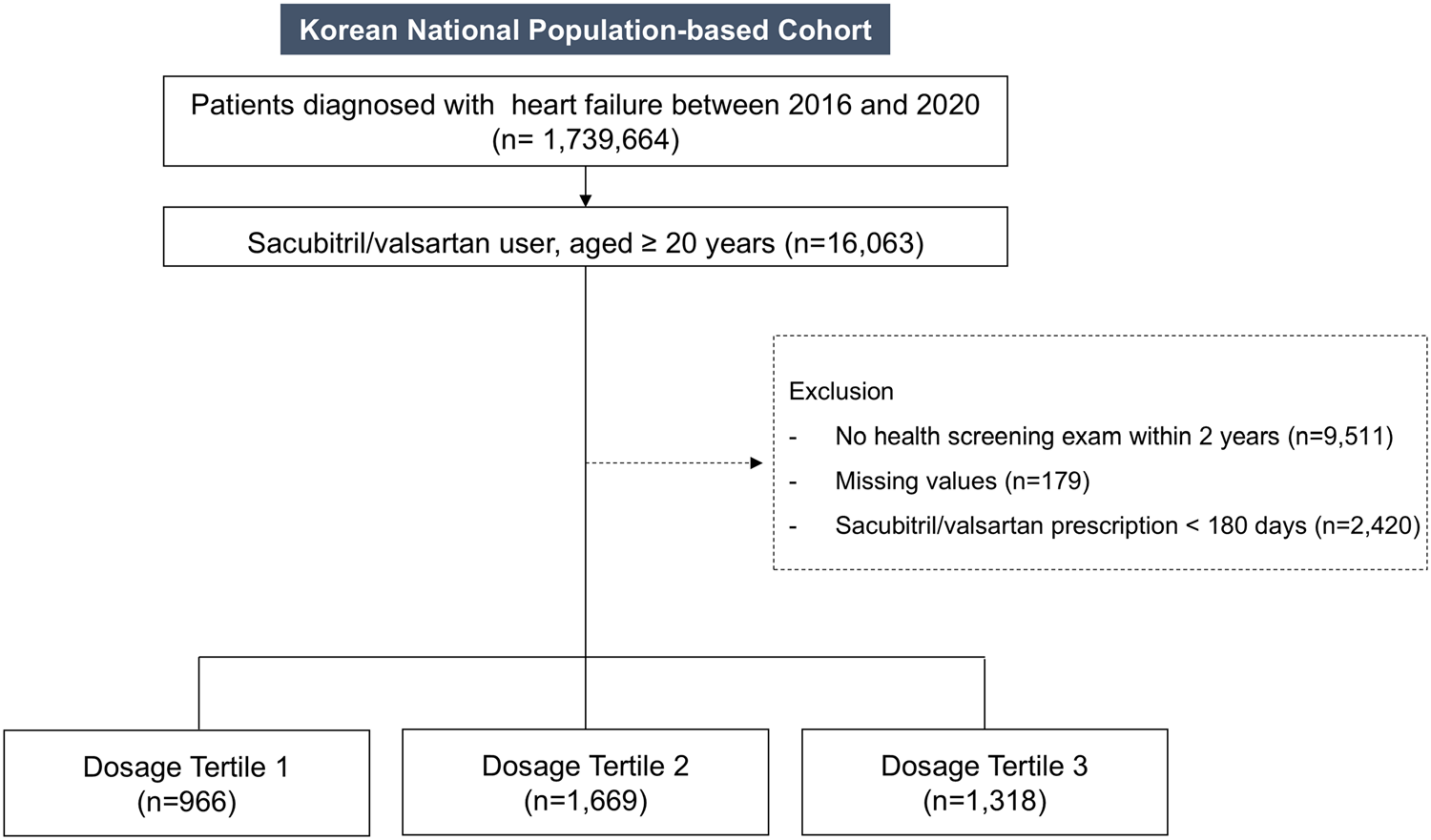
Flowchart of study inclusion

For the study population, we identified adults aged 20 years or older who were diagnosed with HFrEF and prescribed sacubitril/valsartan between 2016 and 2020 (Fig. 1). Sacubitril/valsartan was approved for use in Korea in April 2016, and only symptomatic HFrEF patients with left ventricular ejection fraction of ≤ 40% could be prescribed sacubitril/valsartan and reimbursed in Korea according to federal regulations. The diagnosis of HF was identified based on the International Classification of Diseases, Tenth Revision (ICD-10) codes I50 or I42.0, recorded during an outpatient visit or hospitalization during 2016 to 2020. Patients without health examinations within the 2 years prior to index sacubitril/valsartan prescription and those with missing values in health examination data were excluded. In addition, we only included patients who had been prescribed sacubitril/valsartan for at least 180 consecutive days. Using the prescription database, the average daily dose of sacubitril/valsartan for each patient was calculated by dividing the total prescribed dose by the corresponding prescription duration. The maximum dose of sacubitril/valsartan was 400 mg/day, administered as 97/103 mg twice daily. The study participants were then divided into tertiles according to the average daily dose of sacubitril/valsartan. This approach was selected over fixed-dose groupings to more accurately capture each patient's sacubitril/valsartan exposure over time, as dose adjustments frequently occur in real-world clinical practice.

Comorbidities were defined using diagnostic codes and medications during the past 1 year. The operational definitions of covariates and outcomes are detailed in Supplemental Table S1. The validity of these definitions has been well established in previous studies [14, 15]. De novo HF was defined as no previous diagnosis of HF before the initial HF diagnosis, with a washout period of at least 5 years starting from 2011. Prescription data on HF medication such as beta-blockers, mineralocorticoid receptor antagonists (MRAs), loop diuretics, sodium-glucose cotransporter 2 (SGLT2) inhibitors, ivabradine, aspirin, P2Y12 inhibitors, oral anticoagulants, statins, and calcium-channel blockers were collected in the year of the index date. Notably, only data on SGLT2 inhibitors prescribed for diabetes mellitus were available in the claims database, as SGLT2 inhibitors prescribed for HF are not covered by national insurance in Korea [16, 17]. Definitions of the medications are also provided in Supplemental Table S1.

### Study outcomes and follow-up

The primary outcome was a composite of hospitalization for HF and all-cause mortality. Hospitalization for HF was defined as the first hospitalization event under the ICD-10 codes for HF (I50 and I42.0). All-cause mortality was identified using the official Korean Mortality Database. We also analysed the individual components of the primary outcome as secondary outcomes. The index date was the date of the first sacubitril/valsartan prescription, and the study participants were followed-up until the occurrence of the outcomes or December 31, 2020.

### Statistical analysis

Continuous variables are presented as means ± standard deviations, whereas categorical variables are shown as counts and percentages. Differences between the sacubitril/valsartan dosage groups were assessed using one-way analysis of variance for continuous variables and the chi-square tests for categorical variables.

We employed inverse probability of treatment weighting (IPTW) using stabilized weights derived from propensity scores to compare the outcomes among the three sacubitril/valsartan dosage groups. These scores were calculated using logistic regression, incorporating all baseline characteristics presented in Table 1, such as age, sex, comorbidities, medications for HF and non-HF management, and anthropometric and laboratory measurements. The balance of baseline characteristics across groups was assessed by calculating the maximum absolute standardized difference (ASD), with a maximum ASD of ≤ 0.1 indicating a negligible difference across groups [18]. Weighted incidence rates were determined by dividing the weighted count of outcome events observed during the follow-up period by the sum of the person-time and expressed per 1000 person-years. Kaplan–Meier curves were generated to display weighted incidence probabilities, with statistical comparisons using the log-rank test. The risks in the three groups were evaluated using Cox proportional hazards regression models, with hazard ratios (HRs) for each group presented using the lowest tertile of the sacubitril/valsartan dosage group as a reference. The proportional hazard assumption was verified through graphical inspection of the log-minus-log plots and calculation of Schoenfeld residuals. As a sensitivity analysis, we also conducted multivariable Cox regression analyses in the unweighted study population, adjusting for key variables including age, sex, systolic blood pressure, BMI, hypertension, diabetes mellitus, dyslipidaemia, stroke, intracerebral haemorrhage, atrial fibrillation, myocardial infarction, ischaemic heart disease (IHD), peripheral artery disease, chronic kidney disease, chronic obstructive pulmonary disease (COPD), liver cirrhosis, and cancer. Subgroup analyses stratified by age, sex, BMI, systolic blood pressure, eGFR, de novo or chronic HF, diabetes mellitus, IHD, and atrial fibrillation were conducted using multivariable Cox regression analysis to evaluate potential effect modifications. Statistical significance was set at *p* < 0.05. All analyses were performed using SAS version 9.4 (SAS Institute, Cary, NC, USA).

**Table 1:** Baseline characteristics categorized by average daily dose of sacubitril/valsartan before and after propensity score weighting

	Total (n = 3953)	Propensity Score Weighting									
		Before					After				
		Tertile 1 (n = 966)	Tertile 2 (n = 1669)	Tertile 3 (n = 1318)	P-value	ASD	Tertile 1 (n = 962)	Tertile 2 (n = 1670)	Tertile 3 (n = 1319)	P-value	ASD
Age, years	62.6 ± 12.4	65.3 ± 11.2	63.5 ± 11.7	59.4 ± 13.4	< 0.001	0.476	62.9 ± 12.1	62.6 ± 12.4	62.8 ± 12.6	0.786	0.028
Men (%)	2884 (73.0)	682 (70.6)	1179 (70.6)	1023 (77.6)	< 0.001	0.161	689 (71.7)	1219 (73.0)	953 (72.2)	0.758	0.029
Health examination data											
BMI (kg/m^2^)	25.0 ± 3.9	24.0 ± 3.4	24. 7 ± 3.7	26.0 ± 4.4	< 0.001	0.513	24.9 ± 3.9	25.0 ± 4.1	25.0 ± 3.9	0.867	0.021
eGFR (mL/min/1.73m^2^)	78 ± 27	76 ± 29	78 ± 25	79 ± 26	0.004	0.136	77 ± 29	78 ± 25	77 ± 26	0.710	0.031
DBP (mmHg)	76 ± 12	73 ± 11	75 ± 12	78 ± 13	< 0.001	0.392	76 ± 11	76 ± 12	76 ± 12	0.921	0.016
SBP (mmHg)	124 ± 17	121 ± 16	124 ± 17	127 ± 18	< 0.001	0.363	124 ± 17	124 ± 17	125 ± 17	0.784	0.029
Glucose (mg/dL)	115 ± 41	116 ± 44	115 ± 39	116 ± 41	0.683	0.032	116 ± 43	115 ± 40	116 ± 41	0.956	0.010
Comorbidities (%)											
Hypertension	3699 (93.6)	893 (92.4)	1549 (92.8)	1257 (95.4)	0.005	0.123	899 (93.5)	1564 (93.7)	1241 (94.1)	0.825	0.025
Diabetes mellitus	1369 (34.6)	338 (35.0)	610 (36.6)	421 (31.9)	0.031	0.097	345 (35.8)	573 (34.3)	461 (34.9)	0.733	0.032
Dyslipidaemia	2732 (69.1)	681 (70.5)	1176 (70.5)	875 (66.4)	0.032	0.089	661 (68.8)	1159 (69.4)	922 (69.9)	0.850	0.024
Ischaemic stroke	368 (9.3)	111 (11.5)	155 (9.3)	102 (7.7)	0.010	0.128	90 (9.4)	154 (9.2)	124 (9.4)	0.988	0.006
Haemorrhagic stroke	18 (0.5)	6 (0.6)	5 (0.3)	7 (0.5)	0.439	0.047	5 (0.53)	7 (0.4)	5 (0.4)	0.866	0.019
AF	1323 (33.5)	356 (36.9)	589 (35.3)	378 (28.7)	< 0.001	0.175	321 (33.4)	557 (33.4)	433 (32.8)	0.937	0.01
MI	658 (16.7)	190 (19.7)	287 (17.2)	181 (13.7)	0.001	0.160	164 (17.0)	275 (16.5)	212 (16.0)	0.822	0.026
IHD	2692 (68.1)	700 (72.5)	1123 (67.3)	869 (65.9)	0.003	0.142	659 (68.5)	1136 (68.0)	893 (67.7)	0.915	0.018
PAD	536 (13.6)	156 (16.2)	219 (13.1)	161 (12.2)	0.020	0.113	130 (13.6)	228 (13.6)	181 (13.7)	0.994	0.005
CKD	470 (11.9)	146 (15.1)	183 (11.0)	141 (10.7)	0.002	0.132	116 (12.0)	199 (11.9)	162 (12.3)	0.960	0.010
ESRD	73 (1.9)	21 (2.2)	31 (1.9)	21 (1.6)	0.595	0.043	17 (1.8)	33 (2.0)	27 (2.0)	0.912	0.018
COPD	453 (11.5)	137 (14.2)	195 (11.7)	121 (9.2)	0.001	0.156	111 (11.5)	191 (11.5)	153 (11.6)	0.996	0.003
Liver cirrhosis	57 (1.4)	18 (1.9)	26 (1.6)	13 (1.0)	0.193	0.074	16 (1.6)	23 (1.4)	21 (1.6)	0.862	0.019
Cancer	197 (5.0)	67 (6.9)	81 (4.9)	49 (3.7)	0.002	0.144	48 (5.0)	85 (5.1)	65 (4.9)	0.972	0.009
Heart failure medication (%)											
Beta-blocker	3639 (92.1)	867 (89.8)	1534 (91.9)	1238 (93.9)	0.001	0.153	884 (91.9)	1538 (92.1)	1215 (92.1)	0.986	0.007
MRA	2954 (74.7)	742 (76.8)	1242 (74.4)	970 (73.6)	0.202	0.074	717 (74.6)	1253 (75.0)	980 (74.3)	0.906	0.016
Loop diuretic	3252 (82.3)	826 (85.5)	1371 (82.1)	1055 (80.1)	0.003	0.145	799 (83.1)	1377 (82.5)	1091 (82.7)	0.918	0.017
SGLT2i	314 (7.9)	75 (7.8)	140 (8.4)	99 (7.5)	0.660	0.033	76 (7.9)	130 (7.8)	98 (7.4)	0.912	0.017
Ivabradine	719 (18.2)	169 (17.5)	295 (17.7)	255 (19.4)	0.407	0.048	177 (18. 5)	304 (18.2)	231 (17.5)	0.827	0.024
Other medication (%)											
Aspirin	2252 (57.0)	577 (59.7)	968 (58.0)	707 (53.6)	0.008	0.123	558 (58.0)	954 (57.1)	763 (57.9)	0.873	0.019
P2Y12i	546 (13.8)	149 (15.4)	245 (14.7)	152 (11.5)	0.012	0.114	132 (13.7)	228 (13.7)	185 (14.0)	0.956	0.011
OAC	1294 (32.7)	348 (36.0)	548 (32.8)	398 (30.2)	0.014	0.124	307 (31.9)	546 (32.7)	424 (32.1)	0.909	0.016
Statin	2768 (70.0)	692 (71.6)	1187 (71.1)	889 (67.5)	0.043	0.091	670 (69.6)	1173 (70.3)	933 (70.7)	0.849	0.024
CCB	655 (16.6)	134 (13.9)	228 (13.7)	293 (22.2)	< 0.001	0.225	167 (17.3)	278 (16.7)	220 (16.7)	0.898	0.018

The average daily doses for sacubitril/valsartan dosage tertiles 1 to 3 were as follows: tertile 1, < 24/26 mg twice daily (< 100 mg/day); tertile 2, 24/26–83.8/89 mg twice daily (100–172.8 mg/day); and tertile 3, > 83.8/89 mg twice daily (> 172.8 mg/day)

AF, atrial fibrillation; ASD, absolute standardized difference; BMI, body mass index; CCB, calcium channel blocker; CKD, chronic kidney disease; COPD, chronic obstructive pulmonary disease; DBP, diastolic blood pressure; eGFR, estimated glomerular filtration rate; ESRD, end-stage renal disease; IHD, ischemic heart disease; MRA, mineralocorticoid receptor antagonist; MI, myocardial infarction; OAC, oral anticoagulant; PAD, peripheral artery disease; P2Y12i, P2Y12 inhibitor; SBP, systolic blood pressure; SGLT2i, sodium-glucose cotransporter 2 inhibitor

## Results

### Baseline characteristics of the study population

A total of 3,953 patients with HF (aged ≥ 20 years) who were prescribed sacubitril/valsartan for at least 6 months were included in the study population. The mean age was 62.6 ± 12.4 years, and 73.0% were men (Table 1). The majority of patients were on optimal background therapy for HF, with 92.1% on beta-blockers, 18.2% on ivabradine, 74.7% on MRAs, and 82.3% on loop diuretics.

**Fig. 2:**
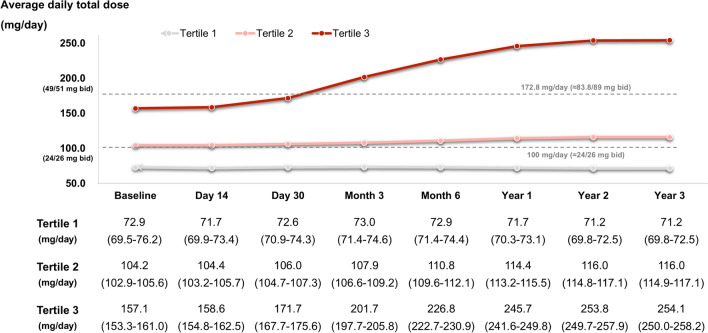
Average daily dose of sacubitril/valsartan throughout the study period across the dosage tertile groups

Patients were divided into tertiles according to their average daily dose of sacubitril/valsartan during the study period, which translates into sacubitril/valsartan dosages of < 24/26 mg twice daily (< 100 mg/day, tertile 1), 24/26–83.8/89 mg twice daily (100–172.8 mg/day, tertile 2), and > 83.8/89 mg twice daily (> 172.8 mg/day, tertile 3). The average daily dose of sacubitril/valsartan for each dosage group during the study period is shown in Fig. 2. The higher dosage groups had higher starting doses, and the tertile 3 group exhibited significant dose up-titration during the 1st year. In contrast, there was no increase in the dose in tertile 1 and a slight increase in tertile 2. Most dose titrations occurred during the 1st year, after which doses remained stable.

Patients in the lower-dose groups were characterized by older age, a higher proportion of women, and a higher prevalence of comorbidities, including diabetes mellitus, ischaemic stroke, atrial fibrillation, myocardial infarction, IHD, peripheral artery disease, chronic kidney disease, COPD, and cancer, and a lower prevalence of hypertension (*p*-value < 0.050 for all). Furthermore, these patients exhibited significantly lower BMI, eGFR, and systolic and diastolic blood pressures (*p*-value < 0.01 for all). The use of MRAs and loop diuretics was higher in the low-dose groups, whereas the use of beta-blockers was slightly higher in the high-dose groups. The use of antiplatelet agents, oral anticoagulants, and statins was slightly higher in the low-dose groups. After IPTW, the three groups were well balanced (ASD < 0.1 for all covariates), and there were no statistically significant differences in baseline characteristics across the dosage groups (Table 1).

### Clinical outcomes according to the average daily dose of sacubitril/valsartan

**Fig. 3:**
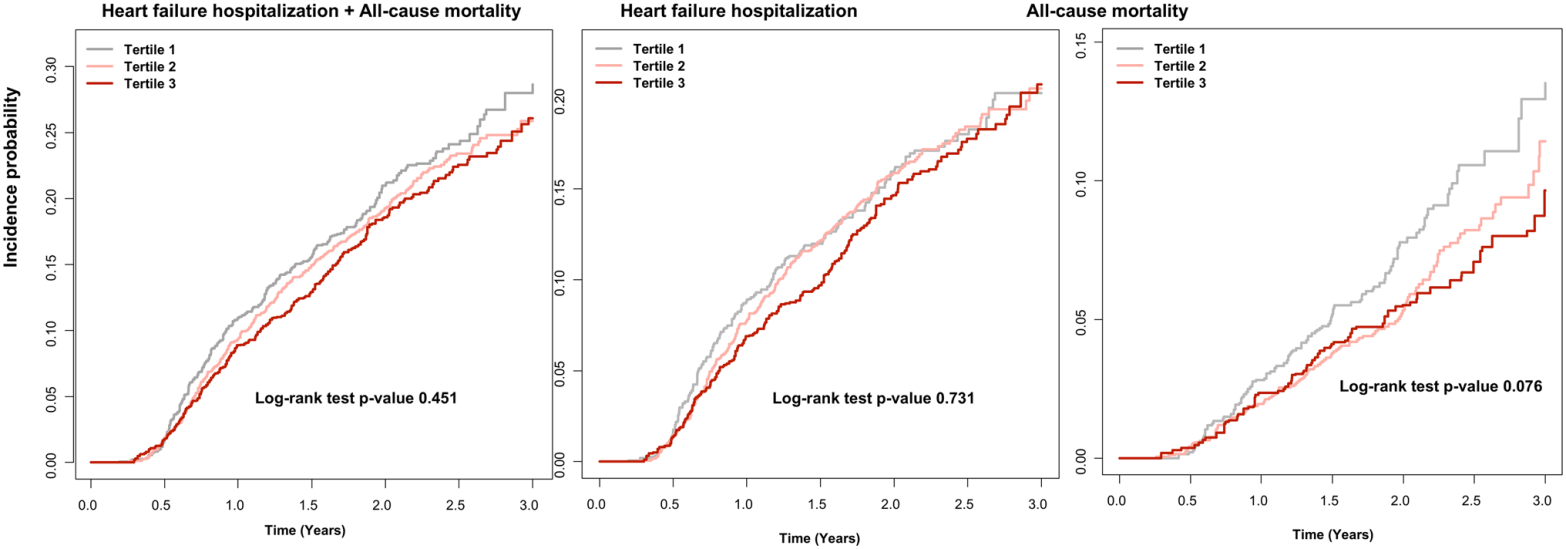
Cumulative incidence probability curves in the propensity score weighted cohort

During a mean follow-up of 2.0 ± 0.7 years, the primary outcome occurred in 808 patients (20.4%). There were 625 hospitalizations for HF (15.8%) and 277 deaths (7.0%). Weighted Kaplan–Meier curves revealed no significant differences in the incidence of the primary outcome or individual outcomes of HF hospitalization and all-cause mortality across the three groups (Fig. 3). However, a non-significant tendency toward lower mortality was observed with higher sacubitril/valsartan doses (log-rank test, *p*-value = 0.076). Cox regression analysis showed no significant difference in the risk of the primary outcome among the three groups (*p*-value = 0.384) (Table 2). Regarding individual outcomes, no significant difference was observed in the risk of hospitalization for HF according to the sacubitril/valsartan dosage. However, there was a significant trend for a lower risk of all-cause mortality in higher dosage groups (*p*-value for trend = 0.019), and patients in the highest dosage group had significantly reduced mortality compared with those in the lowest dosage group (HR 0.69, 95% CI 0.51–0.94).

**Table 2:** Study outcomes

	N	Event	Duration (PY)	Incidence rate (per 1000 PY)	Hazard ratio (95% CI)	P-value	P-for-trend
Composite outcome of hospitalization for heart failure and all-cause mortality							
Tertile 1	962	208.8	1854	112.6	1 (ref.)	0.384	0.169
Tertile 2	1670	339.7	3250	104.5	0.93 (0.78–1.10)		
Tertile 3	1319	271.9	2740	99.3	0.88 (0.74–1.06)		
Hospitalization for heart failure							
Tertile 1	961.6	153.5	1854	82.8	1 (ref.)	0.695	0.437
Tertile 2	1670.1	266.6	3250	82.0	0.99 (0.81–1.21)		
Tertile 3	1319.3	209.0	2740	76.3	0.93 (0.75–1.14)		
All-cause mortality							
Tertile 1	961.6	83.1	1854	41.2	1 (ref.)	0.047	0.019
Tertile 2	1670.1	113.0	3250	31.9	0.77 (0.58–1.02)		
Tertile 3	1319.3	87.8	2740	29.6	0.69 (0.51–0.94)		

The average daily doses for sacubitril/valsartan dosage tertiles 1 to 3 were as follows: tertile 1, < 24/26 mg twice daily (< 100 mg/day); tertile 2, 24/26–83.8/89 mg twice daily (100–172.8 mg/day); and tertile 3, > 83.8/89 mg twice daily (> 172.8 mg/day)

PY, person-years

Consistent results were demonstrated in sensitivity analyses that used multivariable Cox regression models in the unweighted study population (Supplemental Table S2). In the unadjusted models, there were a lower risk of the primary outcome, as well as the individual outcomes of HF hospitalization and all-cause mortality with higher dosage. However, after multivariable adjustment for important clinical variables, there was no significant difference in the risk of primary outcome or hospitalization for HF among the three dosage groups. However, there was a significantly lower risk of mortality in the high-dose groups.

### Subgroup analyses

**Fig. 4:**
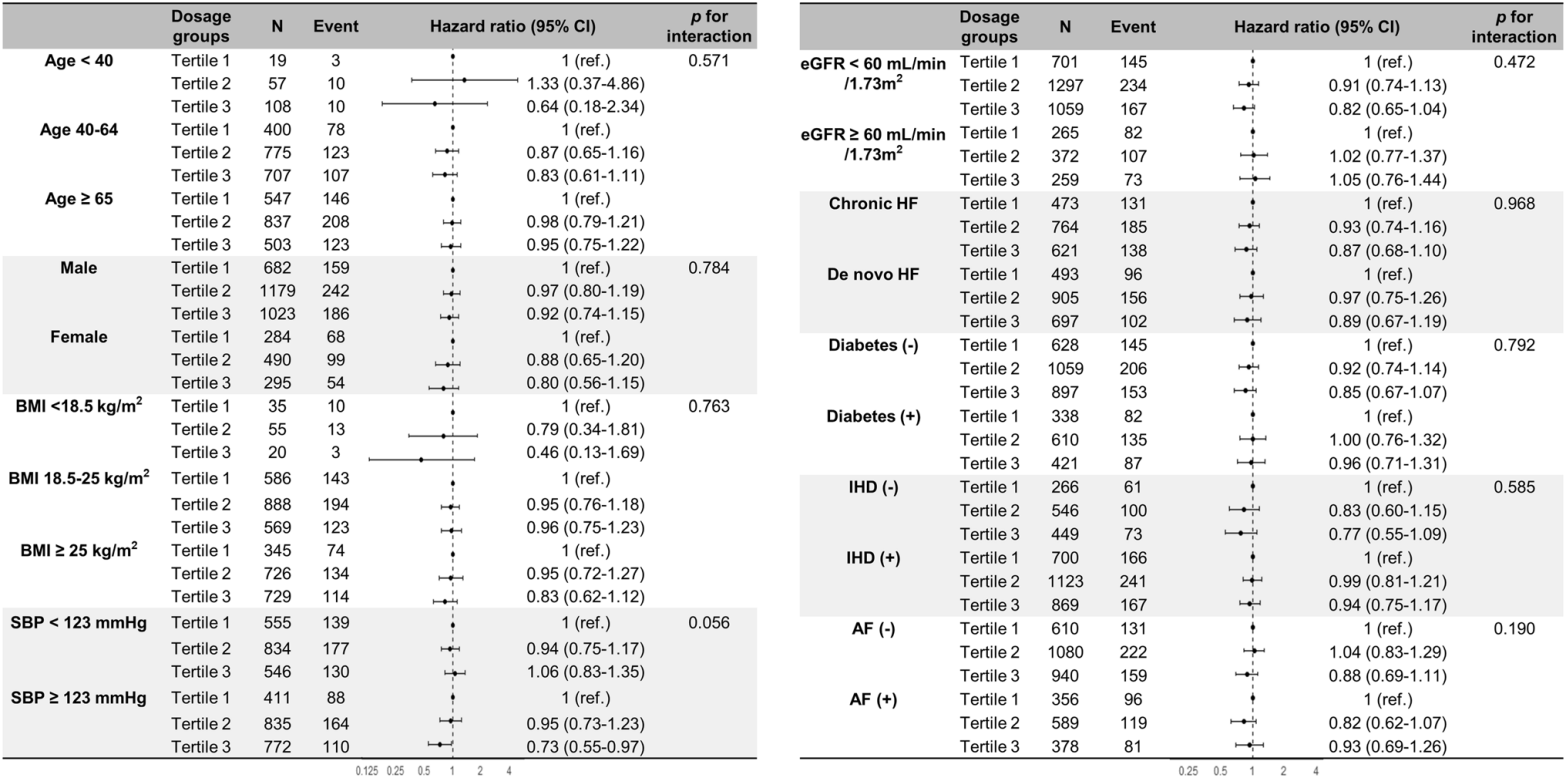
Subgroup analyses for the primary outcome. AF, atrial fibrillation; BMI, body mass index; eGFR, estimated glomerular filtration rate; HF, heart failure; IHD, ischaemic heart disease; SBP, systolic blood pressure

In the subgroup analyses, there were no significant interactions concerning the primary outcome across the three dosage groups when stratified by age, sex, BMI, systolic blood pressure, renal function, de novo or chronic HF, diabetes mellitus, IHD, or atrial fibrillation (Fig. 4, Supplemental Table S3). While there was no difference in HF hospitalization according to sacubitril/valsartan dosage in most subgroups, there was a marginal benefit with the highest tertile of sacubitril/valsartan dosage in patients with systolic blood pressure higher than the median (123 mmHg) (Supplemental Table S4). The tendency for lower all-cause mortality with higher sacubitril/valsartan dose was consistent in most subgroups, except for significant interaction between sacubitril/valsartan dose and eGFR; patients with decreased eGFR (< 60 mL/min/1.73 m^2^) had significantly lower mortality with higher sacubitril/valsartan doses, whereas no benefit with higher sacubitril/valsartan dosage was observed in patients with normal eGFR (≥ 60 mL/min/1.73 m^2^) (Supplemental Table S5).

## Discussion

In this real-world observation based on a nationwide population-based cohort of patients with HFrEF, we demonstrated no significant difference in the composite risk of hospitalization for HF and all-cause mortality across tertiles of the average daily dosage of sacubitril/valsartan over a follow-up of approximately 2 years (Graphical abstract). Patients who received a lower average daily dose of sacubitril/valsartan had more high-risk features, such as older age, lower eGFR, and more comorbidities, excluding hypertension. They also had lower blood pressure and BMI. However, these differences in baseline characteristics were extensively matched using IPTW with propensity scores. Regarding individual outcomes, the risk of hospitalization for HF did not significantly differ among the three groups. At the same time, there was a tendency for lower all-cause mortality in the higher sacubitril/valsartan dosage groups. These results suggest that similar benefits of sacubitril/valsartan in terms of clinical outcomes can be achieved with lower doses, although aiming for higher doses should always be attempted, as lower mortality rates were observed with higher doses.

Sacubitril/valsartan, currently the only available angiotensin receptor–neprilysin inhibitor, has been established as the preferred option over ACEi or angiotensin II receptor blockers (ARB) as one of the four pillars of guideline-directed medical therapy for HFrEF, along with beta-blockers, MRAs, and SGLT2 inhibitors [2, 3]. The target dose of 97/103 mg twice daily (400 mg/day) for sacubitril/valsartan was set in the PARADIGM-HF trial. It was chosen to obtain serum concentrations of valsartan equivalent to valsartan 160 mg twice daily and adequate inhibition of neprilysin at 90% of its maximum capacity [19]. The TITRATION (Safety and Tolerability of Initiating LCZ696 in Heart Failure Patients) study reported that over 80% of patients with chronic HFrEF could achieve the target dose of sacubitril/valsartan through gradual titration over 3 or 6 weeks [20]. However, this study only included patients who successfully completed a five-day run-in period on 24/26 mg of sacubitril/valsartan administered twice daily (totalling 100 mg/day). The TRANSITION (Comparison of Pre- and Post-discharge Initiation of LCZ696 Therapy in HFrEF Patients After an Acute Decompensation Event) study reported that achieving target dose of sacubitril/valsartan is feasible in approximately 50% of HFrEF patients stabilized after an acute HF event; to note, this trial enrolled only patients with a systolic blood pressure of ≥ 110 mmHg [21]. Indeed, fewer patients in real-world practice achieved the target dose of sacubitril/valsartan. with only 25% reaching the target dose 12 months after drug initiation, as reported in a large multicentre registry [8]. Moreover, even 42% of patients enrolled in the PARADIGM-HF trial required dose reduction during the study period, despite the study only enrolling subjects who passed the run-in period with sacubitril/valsartan 97/103 mg twice daily (400 mg/day) [22]. Consequently, the discrepancy between real-world practice and the pivotal clinical trial has raised questions regarding the real-world effectiveness of a lower versus recommended dose of sacubitril/valsartan as well as the optimal sacubitril/valsartan dose in terms of clinical benefit.

Studies regarding the outcomes by different doses of sacubitril/valsartan to date are scarce and predominantly confined to post-hoc analyses of previous trials, each bearing its unique limitations. A *post-hoc* analysis of the PARADIGM-HF trial revealed that the risk of cardiovascular death or HF hospitalization was 2.5 times higher in the group that underwent dose reductions, although it is important to acknowledge that these patients often exhibited more high-risk clinical features, contributing to increased event rates. Indeed, the magnitude of benefit of sacubitril/valsartan did not significantly differ according to doses, when it was compared with patients taking enalapril who underwent a similar degree of dose reductions. However, the PARADIGM-HF study was an RCT composed of individuals in relatively good health who could endure a stringent run-in period. Even patients who underwent dose reductions had tolerated the maximal dose of sacubitril/valsartan for a median of 249 days, thus not fully reflecting the real-world population. In a post-hoc analysis of the PIONEER-HF (Comparison of Sacubitril/Valsartan Versus Enalapril on Effect on NT-proBNP in Patients Stabilized From an Acute Heart Failure Episode) trial, the benefits of sacubitril/valsartan to enalapril across different dose levels did not show significant differences [23]. Yet, this post-hoc analysis was underpowered due to a low number of events in each dose category and a short follow-up duration of 8 weeks. In addition, the PIONEER-HF trial focused on patients hospitalised for acute decompensated HF, thus did not include stable, ambulatory patients with chronic HFrEF [23]. The post hoc analysis of the PROVE-HF (Prospective Study of Biomarkers, Symptom Improvement, and Ventricular Remodeling During Sac/Val Therapy for HF), which included 794 patients with HFrEF, demonstrated similar improvement in surrogate end points, including N-terminal pro–B-type natriuretic peptide (NT-proBNP) levels, Kansas City Cardiomyopathy Questionnaire (KCCQ)-23 scores, and echocardiographic parameters of cardiac reverse remodelling, across sacubitril/valsartan dosage tertiles over 6 months [13]. However, this study did not examine the impact of dose on clinical outcomes. A small observational study also reported similar improvements in NT-proBNP levels, exercise performance, and cardiac remodelling parameters over 1 year between low and high sacubitril/valsartan dosage groups [24]. In contrast, a smaller propensity-matched retrospective cohort of 652 patients reported that 97/103 mg or 49/51 mg sacubitril/valsartan twice daily (totalling 400 or 200 mg/day) was associated with lower mortality or HF hospitalization rates than 24/26 mg sacubitril/valsartan twice daily (100 mg/day) [25]. The results of our study add to the much-needed evidence on the relationship between the sacubitril/valsartan dose and outcomes. Furthermore, consistent results were observed in subgroup analyses, especially after stratification by BMI and de novo or chronic HF, supporting the generalizability of the study results. Contrary to previous controlled trials, no rapid dose escalation was observed in this real-world study, and the average daily doses of sacubitril/valsartan in tertiles 2 and 3 did not reach 50% of the maximal dose. Interestingly, it is noteworthy that the primary outcome remained consistently unaffected. Building upon previous studies, the current study suggests that sacubitril/valsartan offers comparable benefits in clinical outcomes across different dosages, emphasizing the significance of initiating and sustaining sacubitril/valsartan therapy at doses the patient can tolerate. In addition, the current study may help alleviate concerns among physicians over rapidly increasing sacubitril/valsartan doses or the mandatory application of its highest dose, advocating for a more adaptable treatment regimen.

The necessity of reaching and maintaining the same maximal dose of HF medication for every individual has been repeatedly questioned, and multiple studies have reported similar effects with submaximal doses of HF medication in certain populations. Beta-blockers are known to have the most dose-dependent benefit in HFrEF, although there are mixed reports on whether the magnitude of heart rate reduction or the dose of beta-blocker affects outcomes to a greater extent [10, 26]. In the MERIT-HF (Metoprolol CR/XL Randomized Intervention Trial in HF) trial, reduction in mortality and heart rate was similar in the low- and high-dose subgroups, suggesting that a similar level of beta-blockade can be attained with lower doses in certain populations [10]. The low-dose subgroup were older and-had worse symptoms, and lower blood pressure. Meanwhile, SGLT2 inhibitors and MRAs seem to have a dose-independent effect [11, 27, 28]. ACEi/ARBs seem to have small or early-plateauing dose-dependent effects on outcome [29, 30]. Furthermore, a post hoc analysis of BIOSTAT-CHF (The BIOlogy Study to TAilored Treatment in Chronic Heart Failure) suggested that women might fully benefit from ACEi/ARB or beta-blockers at lower doses than men, questioning the true optimal medical therapy in women versus men [12]. These outcomes are supported by the physiological properties of women, such as lower body weight and higher body fat proportion, as well as reduced cardiac, hepatic, and renal functions, all of which contribute to higher plasma levels of both lipophilic and hydrophilic drugs [31]. Similarly, frail patients exhibit increased adiposity, decreased cardiac output, and diminished hepatic and renal clearance, leading to alterations in the pharmacokinetics and pharmacodynamics [32]. Taken together, these findings suggest that the optimal dose of HFrEF medications might vary among individuals and support the observation in the current study that the effectiveness of sacubitril/valsartan may not significantly differ across different doses if a maximally tolerated dose, which can be variable for each patient, is administered. Although lower mortality rates were observed at higher doses in the current study, it should be noted that there is a possibility of residual confounding related to unadjusted high-risk clinical features in the lower-dose groups, such as left ventricular EF, frailty or socioeconomic status, which may have influenced the observed differences in mortality.

### Strengths and limitations

To our knowledge, this is the largest study to date, utilizing a nationwide database of nearly 4000 patients with HFrEF to examine the relationship between sacubitril/valsartan dose and outcomes. Strengths include a comprehensive database encompassing the entire Korean population with complete data on prescriptions and outcomes, including only patients who were prescribed sacubitril/valsartan for at least 6 months, and calculating the average daily dose throughout the study period instead of using only the initial or final dose. However, some limitations should be noted. First of all, due to the nature of the claims database, some important variables, including echocardiographic parameters, such as left ventricular EF, E/e’, cardiac biomarkers, symptoms, and frailty, were not available for analysis, which may have resulted in residual bias not accounted for in the IPTW. In addition, we were unable to ascertain the specific reasons for sub-maximal sacubitril/valsartan dosing, such as intolerance due to renal dysfunction, hypotension, patient noncompliance, or physician-related factors. Likewise, it was not possible to include a comparable HFrEF population taking ACEi/ARBs from the NHIS database, because unlike sacubitril/valsartan there are no restrictions in regard to left ventricular ejection fraction in prescription of ACEi/ARBs and therefore these drugs are prescribed to a more heterogeneous population including hypertension and HF with preserved or moderately reduced ejection fraction. Second, although we took meticulous care to control for confounders and ensured that the covariates were well-balanced after IPTW, residual confounding due to unmeasured or unknown variables may still influence the outcomes. Third, the doses of other medications for HFrEF were not taken into account. Additionally, data on prescribed SGLT2 inhibitors were not fully captured because SGLT2 inhibitors were not reimbursed for HF without diabetes in Korea during the study period. Although no significant difference was observed in the subgroup of diabetic patients for whom SGLT2 inhibitors were reimbursed, future studies including these data will be needed. Fourth, data on follow-up measurements of blood pressure were not available. Finally, some HF-related clinical outcomes, such as heart transplantation or implantation of a left ventricular assist device, were not assessed; however, these events would have been included in hospitalization for HF.

### Conclusions

No significant differences were observed in the composite outcomes of hospitalization for HF and all-cause mortality across the different sacubitril/valsartan dosage groups. Submaximal doses of sacubitril/valsartan can be sufficient to achieve clinical benefits. These results support the idea of an individualized dose-titration regimen guided by patient tolerability rather than fixed target doses.

## Supplementary Information

Below is the link to the electronic supplementary material.

Supplementary file 1 (DOCX 99 KB)

## Data Availability

Raw data, sample cohort data, or customized cohort data are all available from designated terminals if approved by the NHIS. Those unauthorized are restricted from accessing the data.
