# Supplementary material for: Clinical outcomes according to the average daily dose of sacubitril/valsartan: a nationwide longitudinal cohort study
Source: Clin Res Cardiol. 2025 Mar 18;115(9):1455–66. doi: 10.1007/s00392-025-02602-x (PMC13457236; doi:10.1007/s00392-025-02602-x)
Supplement: Supplementary file 1 [file 392_2025_2602_MOESM1_ESM.docx]

**Supplemental Appendix**

**Clinical outcomes according to the Average Daily Dose of Sacubitril/valsartan:
A nationwide longitudinal cohort study**

**Table of Contents**

Page 2-3 Supplemental Table 1	Definitions of variables and outcomes

Page 4-5 Supplemental Table 2	Sensitivity analysis: Study outcomes using multivariable-adjusted Cox proportional hazards models from the unweighted study population

Page 6-9 Supplemental Table 3	The risk of the primary outcome according to various subgroups.

Page 10-13 Supplemental Table 4	The risk of heart failure hospitalisation according to various subgroups in multivariable Cox regression analyses.

Page 14-18 Supplemental Table 5	The risk of all-cause mortality according to various subgroups in multivariable Cox regression analyses.

**Supplemental Table 1. Definitions of variables and outcomes.**

| **Variables** | **ICD-10-CM codes** | **Additional definitions** |
| --- | --- | --- |
| *Inclusion criteria* |  |  |
| Heart failure (including DCM) | I50, I42.0 (DCM) | Admission ≥ 1 or outpatient clinic ≥ 1 |
| *Covariates^*^* |  |  |
| Hypertension | I10-I13, I15 | Admission ≥ 1 or outpatient clinic ≥ 2 Prescription of one or more anti-hypertensive drugs (angiotensin-converting enzyme inhibitor, angiotensin II receptor blocker, calcium-channel blocker, hydrochlorothiazide, indapamide, chlorthalidone, beta-blockers) |
| Diabetes mellitus | E10-E14 | Admission ≥ 1 or outpatient clinic ≥ 2 Prescription of one or more anti-diabetic drugs (metformin, sulfonylureas, meglitinides, thiazolidinediones, dipeptidyl peptidase-4 inhibitors, α-glucosidase inhibitors, SGLT2-inhibitor, GLP-1 agonist, or insulin) |
| Dyslipidaemia | E78 | Admission ≥ 1 or outpatient clinic ≥ 2 Prescription of one or more lipid-lowering medication (statin, ezetimibe, fenofibrate) |
| Ischemic stroke | I63, I64 | Admission ≥ 1 or outpatient clinic ≥ 2 |
| Haemorrhagic stroke | I60-62 | Admission ≥ 1 or outpatient clinic ≥ 2 |
| Atrial fibrillation | I48 | Admission ≥ 1 or outpatient clinic ≥ 2 |
| Myocardial infarction | I21, I22 | Admission ≥ 1 or outpatient clinic ≥ 2 |
| Peripheral artery disease | I70.2, I73 | Admission ≥ 1 or outpatient clinic ≥ 2 |
| Chronic kidney disease | N00-007, N11, I12, N18-19, Q61 | Admission ≥ 1 or outpatient clinic ≥ 2 |
| End-stage renal disease | N18, N19, Z49, Z94.0, Z99.2 | 1) Dialysis ≥ 2 [Procedure codes: O7011-7020 (haemodialysis), O7071-O7075 (peritoneal dialysis)]  or 2) Registration for rare disease [V001 (haemodialysis), V003 (peritoneal dialysis)] |
| COPD | J43-44 | Admission ≥ 1 or outpatient clinic ≥ 2 |
| Liver cirrhosis | K70.3, K74.3-74.6, K71.7, K76.1 | Admission ≥ 1 or outpatient clinic ≥ 2 |
| Cancer | C00-97 | Admission ≥ 1 or outpatient clinic ≥ 1  Registration for rare disease [V193 (cancer)] |
| *Medications* |  |  |
| Beta-blockers |  | Carvedilol, bisoprolol, nebivolol, metoprolol |
| MRA^†^ |  | Spironolactone |
| Loop diuretics |  | Furosemide, torsemide |
| SGLT2i |  | Dapagliflozin, empagliflozin |
| Ivabradine |  |  |
| Aspirin |  |  |
| P2Y12i |  | Clopidogrel, prasugrel, ticagrelor |
| Oral anticoagulant |  | Warfarin, rivaroxaban, dabigatran, apixaban, edoxaban |
| Statin |  | Atorvastatin, rosuvastatin, fluvastatin, lovastatin, pravastatin, simvastatin, pitavastatin |
| Calcium-channel blocker |  | Amlodipine, nifedipine, cilnidipine, felodipine, lacidipine, lercanidipine, manidipine, nicardipine |
| *Endpoints* |  |  |
| Heart failure hospitalisation | I50, I42.0 (DCM) | Admission for heart failure as a primary diagnosis ≥ 1 |

*Covariates were defined by diagnoses during hospitalisation or at outpatient clinic during the past 1 year.

^†^Eplerenone was not available in Korea during the study period, as it was only approved by the Korean FDA after October 2020.

Abbreviations: COPD, chronic obstructive pulmonary disease; CT, computed tomography; DCM, dilated cardiomyopathy; GLP-1, Glucagon-like peptide-1; ICD-10, International Classification of Diseases 10^th^ revision from the World Health Organization; MRA, mineralocorticoid receptor antagonist; MRI, magnetic resonance imaging; P2Y12i, P2Y12 inhibitor; SGLT2, sodium-glucose cotransporter-2 inhibitor

**Supplemental Table 2. Sensitivity analysis: Study outcomes using multivariable-adjusted Cox proportional hazards models from the unweighted study population.**

|  | **N** | **Event** | **IR (/per 1,000 PY)** | **Unadjusted model** | **Model 1** | **Model 2** | **Model 3** |
| --- | --- | --- | --- | --- | --- | --- | --- |
| **Composite outcome of hospitalisation for heart failure and all-cause mortality** | | | | | | | |
| Tertile 1 | 966 | 227 | 123.1 | 1 (ref.) | 1 (ref.) | 1 (ref.) | 1 (ref.) |
| Tertile 2 | 1669 | 341 | 104.8 | 0.85 (0.72-1.01) | 0.88 (0.75-1.05) | 0.91 (0.77-1.07) | 0.95 (0.80-1.12) |
| Tertile 3 | 1318 | 240 | 87.1 | 0.71 (0.59-0.85) | 0.79 (0.66-0.95) | 0.83 (0.69-1.00) | 0.89 (0.73-1.07) |
|  |  |  |  | *P*-for-trend <0.001 | *P*-for-trend = 0.012 | *P*-for-trend = 0.053 | *P*-for-trend = 0.215 |
| **HF hospitalisation** | | | | | | | |
| Tertile 1 | 966 | 165 | 89.5 | 1 (ref.) | 1 (ref.) | 1 (ref.) | 1 (ref.) |
| Tertile 2 | 1669 | 270 | 82.9 | 0.93 (0.76-1.13) | 0.96 (0.79-1.16) | 0.99 (0.81-1.20) | 1.02 (0.84-1.24) |
| Tertile 3 | 1318 | 190 | 68.9 | 0.77 (0.63-0.95) | 0.844 (0.68-1.04) | 0.90 (0.73-1.12) | 0.95 (0.76-1.18) |
|  |  |  |  | *P*-for-trend = 0.013 | *P*-for-trend = 0.109 | *P*-for-trend = 0.339 | *P*-for-trend = 0.622 |
| **All-cause mortality** | | | | | | | |
| Tertile 1 | 966 | 96 | 47.7 | 1 (ref.) | 1 (ref.) | 1 (ref.) | 1 (ref.) |
| Tertile 2 | 1669 | 111 | 31.2 | 0.65 (0.49-0.85) | 0.69 (0.53-0.91) | 0.69 (0.53-0.91) | 0.73 (0.56-0.96) |
| Tertile 3 | 1318 | 70 | 23.7 | 0.48 (0.35-0.65) | 0.58 (0.42-0.79) | 0.59 (0.43-0.81) | 0.63 (0.46-0.87) |
|  |  |  |  | *P*-for-trend <0.001 | *P*-for-trend < 0.001 | *P*-for-trend < 0.001 | *P*-for-trend = 0.004 |

Model 1 adjusted for age and sex. Model 2 adjusted for age, sex, systolic blood pressure, and body mass index. Finally, model 3 adjusted for all the variables in model 2 as well as comorbidities of hypertension, diabetes, dyslipidaemia, ischemic stroke, intracerebral haemorrhage, atrial fibrillation, myocardial infarction, ischemic heart disease, peripheral artery disease, chronic kidney disease, chronic obstructive pulmonary disease, liver cirrhosis, and cancer.

Abbreviations: IR, incidence rate; HF, heart failure

**Supplemental Table 3. The risk of the primary outcome according to various subgroups.**

|  | **Dosage groups** | **N** | **Event** | **IR (per 1,000 PY)** | **Unadjusted model** | **Model 1** | **Model 2** | **Model 3** |
| --- | --- | --- | --- | --- | --- | --- | --- | --- |
| **Age < 40** | **Tertile 1** | 19 | 3 | 80.3 | 1 (ref.) | 1 (ref.) | 1 (ref.) | 1 (ref.) |
|  | **Tertile 2** | 57 | 10 | 93.2 | 1.16 (0.32-4.21) | 1.13 (0.31-4.09) | 1.23 (0.34-4.46) | 1.33 (0.37-4.86) |
|  | **Tertile 3** | 108 | 10 | 44.9 | 0.55 (0.15-1.99) | 0.54 (0.15-1.96) | 0.63 (0.17-2.29) | 0.64 (0.18-2.34) |
| **Age 40-64** | **Tertile 1** | 400 | 78 | 98.4 | 1 (ref.) | 1 (ref.) | 1 (ref.) | 1 (ref.) |
|  | **Tertile 2** | 775 | 123 | 79.4 | 0.81 (0.61-1.07) | 0.81 (0.61-1.08) | 0.82 (0.62-1.10) | 0.87 (0.65-1.16) |
|  | **Tertile 3** | 707 | 107 | 70.5 | 0.72 (0.53-0.96) | 0.74 (0.55-0.99) | 0.77 (0.57-1.04) | 0.83 (0.61-1.11) |
| **Age ≥ 65** | **Tertile 1** | 547 | 146 | 143.9 | 1 (ref.) | 1 (ref.) | 1 (ref.) | 1 (ref.) |
|  | **Tertile 2** | 837 | 208 | 130.2 | 0.90 (0.73-1.12) | 0.91 (0.74-1.13) | 0.94 (0.76-1.17) | 0.98 (0.79-1.21) |
|  | **Tertile 3** | 503 | 123 | 121.1 | 0.84 (0.66-1.06) | 0.84 (0.66-1.07) | 0.89 (0.70-1.13) | 0.95 (0.75-1.22) |
|  |  |  |  |  | *P*-interaction = 0.601 | *P*-interaction = 0.634 | *P*-interaction = 0.654 | *P*-interaction = 0.571 |
| **Male** | **Tertile 1** | 682 | 159 | 122.2 | 1 (ref.) | 1 (ref.) | 1 (ref.) | 1 (ref.) |
|  | **Tertile 2** | 1179 | 242 | 105.4 | 0.86 (0.71-1.05) | 0.91 (0.74-1.11) | 0.93 (0.76-1.14) | 0.97 (0.80-1.19) |
|  | **Tertile 3** | 1023 | 186 | 87.2 | 0.71 (0.58-0.88) | 0.81 (0.66-1.01) | 0.86 (0.69-1.07) | 0.92 (0.74-1.15) |
| **Female** | **Tertile 1** | 284 | 68 | 125.2 | 1 (ref.) | 1 (ref.) | 1 (ref.) | 1 (ref.) |
|  | **Tertile 2** | 490 | 99 | 103.3 | 0.83 (0.61-1.12) | 0.83 (0.61-1.13) | 0.85 (0.62-1.15) | 0.88 (0.65-1.20) |
|  | **Tertile 3** | 295 | 54 | 86.6 | 0.69 (0.48-0.98) | 0.73 (0.51-1.04) | 0.76 (0.53-1.09) | 0.80 (0.56-1.15) |
|  |  |  |  |  | *P*-interaction = 0.974 | *P*-interaction = 0.837 | *P*-interaction = 0.821 | *P*-interaction = 0.784 |
| **BMI <18.5 kg/m^2^** | **Tertile 1** | 35 | 10 | 157.1 | 1 (ref.) | 1 (ref.) | 1 (ref.) | 1 (ref.) |
|  | **Tertile 2** | 55 | 13 | 126.0 | 0.79 (0.35-1.80) | 0.75 (0.33-1.71) | 0.74 (0.32-1.69) | 0.79 (0.34-1.81) |
|  | **Tertile 3** | 20 | 3 | 70.6 | 0.44 (0.12-1.59) | 0.44 (0.12-1.59) | 0.45 (0.12-1.63) | 0.46 (0.13-1.69) |
| **BMI ≥ 18.5 and <25 kg/m^2^** | **Tertile 1** | 586 | 143 | 129.1 | 1 (ref.) | 1 (ref.) | 1 (ref.) | 1 (ref.) |
|  | **Tertile 2** | 888 | 194 | 111.5 | 0.86 (0.70-1.07) | 0.89 (0.71-1.10) | 0.91 (0.73-1.12) | 0.95 (0.76-1.18) |
|  | **Tertile 3** | 569 | 123 | 104.1 | 0.80 (0.63-1.02) | 0.86 (0.68-1.09) | 0.89 (0.70-1.14) | 0.96 (0.75-1.23) |
| **BMI ≥ 25 kg/m^2^** | **Tertile 1** | 345 | 74 | 110.0 | 1 (ref.) | 1 (ref.) | 1 (ref.) | 1 (ref.) |
|  | **Tertile 2** | 726 | 134 | 94.8 | 0.86 (0.65-1.15) | 0.91 (0.68-1.21) | 0.92 (0.69-1.22) | 0.95 (0.72-1.27) |
|  | **Tertile 3** | 729 | 114 | 74.4 | 0.68 (0.50-0.90) | 0.77 (0.57-1.03) | 0.79 (0.59-1.06) | 0.83 (0.62-1.12) |
|  |  |  |  |  | *P*-interaction = 0.754 | *P*-interaction = 0.807 | *P*-interaction = 0.808 | *P*-interaction = 0.763 |
| **SBP < 123 mmHg (median)** | **Tertile 1** | 555 | 139 | 130.9 | 1 (ref.) | 1 (ref.) | 1 (ref.) | 1 (ref.) |
|  | **Tertile 2** | 834 | 177 | 108.7 | 0.83 (0.67-1.04) | 0.88 (0.70-1.10) | 0.88 (0.71-1.10) | 0.94 (0.75-1.17) |
|  | **Tertile 3** | 546 | 130 | 113.7 | 0.87 (0.68-1.10) | 0.97 (0.77-1.24) | 0.98 (0.77-1.25) | 1.06 (0.83-1.35) |
| **SBP ≥ 123 mmHg (median)** | **Tertile 1** | 411 | 88 | 112.5 | 1 (ref.) | 1 (ref.) | 1 (ref.) | 1 (ref.) |
|  | **Tertile 2** | 835 | 164 | 100.8 | 0.90 (0.69-1.16) | 0.92 (0.71-1.19) | 0.92 (0.71-1.20) | 0.95 (0.73-1.23) |
|  | **Tertile 3** | 772 | 110 | 68.2 | 0.60 (0.46-0.80) | 0.69 (0.52-0.91) | 0.69 (0.52-0.92) | 0.73 (0.55-0.97) |
|  |  |  |  |  | *P*-interaction = 0.029 | *P*-interaction = 0.047 | *P*-interaction = 0.049 | *P*-interaction = 0.056 |
| **eGFR < 60 mL/min/1.73m^2^** | **Tertile 1** | 701 | 145 | 108.8 | 1 (ref.) | 1 (ref.) | 1 (ref.) | 1 (ref.) |
|  | **Tertile 2** | 1297 | 234 | 91.3 | 0.84 (0.68-1.03) | 0.87 (0.70-1.07) | 0.89 (0.72-1.09) | 0.91 (0.74-1.13) |
|  | **Tertile 3** | 1059 | 167 | 73.9 | 0.68 (0.54-0.84) | 0.75 (0.60-0.93) | 0.79 (0.63-0.99) | 0.82 (0.65-1.04) |
| **eGFR ≥ 60 mL/min/1.73m^2^** | **Tertile 1** | 265 | 82 | 160.4 | 1 (ref.) | 1 (ref.) | 1 (ref.) | 1 (ref.) |
|  | **Tertile 2** | 372 | 107 | 154.5 | 0.97 (0.73-1.29) | 0.98 (0.73-1.30) | 1.00 (0.75-1.33) | 1.02 (0.77-1.37) |
|  | **Tertile 3** | 259 | 73 | 147.4 | 0.92 (0.67-1.27) | 0.95 (0.70-1.31) | 1.00 (0.73-1.37) | 1.05 (0.76-1.44) |
|  |  |  |  |  | *P*-interaction = 0.282 | *P*-interaction = 0.463 | *P*-interaction = 0.492 | *P*-interaction = 0.472 |
| **Chronic HF** | **Tertile 1** | 473 | 131 | 143.6 | 1 (ref.) | 1 (ref.) | 1 (ref.) | 1 (ref.) |
|  | **Tertile 2** | 764 | 185 | 123.0 | 0.86 (0.69-1.07) | 0.91 (0.72-1.13) | 0.92 (0.73-1.15) | 0.93 (0.74-1.16) |
|  | **Tertile 3** | 621 | 138 | 104.1 | 0.72 (0.57-0.92) | 0.80 (0.63-1.01) | 0.83 (0.65-1.05) | 0.87 (0.68-1.10) |
| **De novo HF** | **Tertile 1** | 493 | 96 | 103.0 | 1 (ref.) | 1 (ref.) | 1 (ref.) | 1 (ref.) |
|  | **Tertile 2** | 905 | 156 | 89.1 | 0.86 (0.67-1.11) | 0.88 (0.68-1.14) | 0.90 (0.70-1.17) | 0.97 (0.75-1.26) |
|  | **Tertile 3** | 697 | 102 | 71.3 | 0.69 (0.52-0.91) | 0.78 (0.59-1.04) | 0.82 (0.62-1.09) | 0.89 (0.67-1.19) |
|  |  |  |  |  | *P*-interaction = 0.950 | *P*-interaction = 0.989 | *P*-interaction = 0.995 | *P*-interaction = 0.968 |
| **Diabetes (-)** | **Tertile 1** | 628 | 145 | 120.8 | 1 (ref.) | 1 (ref.) | 1 (ref.) | 1 (ref.) |
|  | **Tertile 2** | 1059 | 206 | 99.3 | 0.82 (0.66-1.01) | 0.87 (0.70-1.07) | 0.89 (0.72-1.10) | 0.92 (0.74-1.14) |
|  | **Tertile 3** | 897 | 153 | 80.8 | 0.67 (0.53-0.84) | 0.76 (0.61-0.96) | 0.81 (0.64-1.02) | 0.85 (0.67-1.07) |
| **Diabetes (+)** | **Tertile 1** | 338 | 82 | 127.4 | 1 (ref.) | 1 (ref.) | 1 (ref.) | 1 (ref.) |
|  | **Tertile 2** | 610 | 135 | 114.2 | 0.90 (0.68-1.18) | 0.91 (0.69-1.20) | 0.94 (0.71-1.23) | 1.00 (0.76-1.32) |
|  | **Tertile 3** | 421 | 87 | 100.8 | 0.79 (0.58-1.07) | 0.84 (0.62-1.14) | 0.89 (0.65-1.20) | 0.96 (0.71-1.31) |
|  |  |  |  |  | *P*-interaction = 0.680 | *P*-interaction = 0.885 | *P*-interaction = 0.886 | *P*-interaction = 0.792 |
| **IHD (-)** | **Tertile 1** | 266 | 61 | 119.0 | 1 (ref.) | 1 (ref.) | 1 (ref.) | 1 (ref.) |
|  | **Tertile 2** | 546 | 100 | 92.3 | 0.77 (0.56-1.06) | 0.81 (0.59-1.11) | 0.83 (0.60-1.14) | 0.83 (0.60-1.15) |
|  | **Tertile 3** | 449 | 73 | 76.3 | 0.64 (0.46-0.90) | 0.73 (0.52-1.03) | 0.77 (0.55-1.08) | 0.77 (0.55-1.09) |
| **IHD (+)** | **Tertile 1** | 700 | 166 | 124.6 | 1 (ref.) | 1 (ref.) | 1 (ref.) | 1 (ref.) |
|  | **Tertile 2** | 1123 | 241 | 111.0 | 0.89 (0.73-1.09) | 0.92 (0.75-1.12) | 0.94 (0.77-1.15) | 0.99 (0.81-1.21) |
|  | **Tertile 3** | 869 | 167 | 92.8 | 0.74 (0.60-0.92) | 0.82 (0.66-1.01) | 0.86 (0.69-1.07) | 0.94 (0.75-1.17) |
|  |  |  |  |  | *P*-interaction = 0.710 | *P*-interaction = 0.787 | *P*-interaction = 0.776 | *P*-interaction = 0.585 |
| **AF (-)** | **Tertile 1** | 610 | 131 | 112.1 | 1 (ref.) | 1 (ref.) | 1 (ref.) | 1 (ref.) |
|  | **Tertile 2** | 1080 | 222 | 105.2 | 0.94 (0.75-1.16) | 0.98 (0.79-1.22) | 1.00 (0.81-1.24) | 1.04 (0.83-1.29) |
|  | **Tertile 3** | 940 | 159 | 80.1 | 0.71 (0.56-0.90) | 0.79 (0.63-1.00) | 0.84 (0.66-1.06) | 0.88 (0.69-1.11) |
| **AF (+)** | **Tertile 1** | 356 | 96 | 142.0 | 1 (ref.) | 1 (ref.) | 1 (ref.) | 1 (ref.) |
|  | **Tertile 2** | 589 | 119 | 104.0 | 0.73 (0.56-0.96) | 0.75 (0.58-0.98) | 0.77 (0.59-1.01) | 0.82 (0.62-1.07) |
|  | **Tertile 3** | 378 | 81 | 105.0 | 0.74 (0.55-0.99) | 0.82 (0.61-1.11) | 0.87 (0.64-1.17) | 0.93 (0.69-1.26) |
|  |  |  |  |  | *P*-interaction = 0.203 | *P*-interaction = 0.164 | *P*-interaction = 0.169 | *P*-interaction = 0.190 |

The mean daily doses of sacubitril/valsartan were: tertile 1, <24/26 mg twice daily (<100 mg/day); tertile 2, 24/26–83.8/89 mg twice daily (100–172.8 mg/day); and tertile 3, >83.8/89 mg twice daily (>172.8 mg/day).

Model 1 adjusted for age and sex. Model 2 adjusted for age, sex, systolic blood pressure, and body mass index. Finally, model 3 adjusted for all the variables in model 2 as well as comorbidities of hypertension, diabetes, dyslipidaemia, ischemic stroke, intracerebral haemorrhage, atrial fibrillation, myocardial infarction, ischemic heart disease, peripheral artery disease, chronic kidney disease, chronic obstructive pulmonary disease, liver cirrhosis, and cancer.

Abbreviations: BMI, body mass index; CKD, chronic kidney disease; IHD, ischemic heart disease; IR, incidence rate; HF, heart failure; PY, person-year; SBP, systolic blood pressure

**Supplemental Table 4. The risk of heart failure hospitalisation according to various subgroups in multivariable Cox regression analyses.**

|  | **Dosage groups** | **N** | **Event** | **IR (per 1,000 PY)** | **Unadjusted model** | **Model 1** | **Model 2** | **Model 3** |
| --- | --- | --- | --- | --- | --- | --- | --- | --- |
| **Age < 40** | **Tertile 1** | 19 | 3 | 80.3 | 1 (ref.) | 1 (ref.) | 1 (ref.) | 1 (ref.) |
|  | **Tertile 2** | 57 | 8 | 74.6 | 0.92 (0.24-3.47) | 0.91 (0.24-3.42) | 1.02 (0.27-3.85) | 1.07 (0.28-4.06) |
|  | **Tertile 3** | 108 | 8 | 35.9 | 0.44 (0.12-1.65) | 0.43 (0.12-1.64) | 0.51 (0.14-1.94) | 0.51 (0.14-1.95) |
| **Age 40-64** | **Tertile 1** | 400 | 55 | 69.4 | 1 (ref.) | 1 (ref.) | 1 (ref.) | 1 (ref.) |
|  | **Tertile 2** | 775 | 101 | 65.2 | 0.94 (0.68-1.30) | 0.94 (0.68-1.31) | 0.95 (0.69-1.32) | 0.99 (0.71-1.37) |
|  | **Tertile 3** | 707 | 94 | 62.0 | 0.90 (0.64-1.25) | 0.91 (0.65-1.27) | 0.95 (0.68-1.34) | 1.01 (0.72-1.41) |
| **Age ≥ 65** | **Tertile 1** | 547 | 107 | 105.5 | 1 (ref.) | 1 (ref.) | 1 (ref.) | 1 (ref.) |
|  | **Tertile 2** | 837 | 161 | 100.8 | 0.96 (0.75-1.22) | 0.96 (0.75-1.23) | 1.01 (0.79-1.29) | 1.04 (0.81-1.33) |
|  | **Tertile 3** | 503 | 88 | 86.6 | 0.82 (0.62-1.09) | 0.83 (0.62-1.09) | 0.89 (0.67-1.19) | 0.94 (0.71-1.25) |
|  |  |  |  |  | *P*-interaction = 0.702 | *P*-interaction = 0.684 | *P*-interaction = 0.718 | *P*-interaction = 0.662 |
| **Male** | **Tertile 1** | 682 | 111 | 85.3 | 1 (ref.) | 1 (ref.) | 1 (ref.) | 1 (ref.) |
|  | **Tertile 2** | 1179 | 189 | 82.3 | 0.97 (0.76-1.22) | 1.01 (0.80-1.27) | 1.04 (0.82-1.32) | 1.07 (0.85-1.36) |
|  | **Tertile 3** | 1023 | 150 | 70.3 | 0.83 (0.65-1.06) | 0.92 (0.72-1.18) | 0.98 (0.76-1.27) | 1.04 (0.81-1.34) |
| **Female** | **Tertile 1** | 284 | 54 | 99.4 | 1 (ref.) | 1 (ref.) | 1 (ref.) | 1 (ref.) |
|  | **Tertile 2** | 490 | 81 | 84.5 | 0.85 (0.60-1.20) | 0.85 (0.60-1.20) | 0.89 (0.63-1.25) | 0.91 (0.64-1.29) |
|  | **Tertile 3** | 295 | 40 | 64.2 | 0.65 (0.43-0.97) | 0.68 (0.45-1.02) | 0.72 (0.48-1.08) | 0.75 (0.50-1.13) |
|  |  |  |  |  | *P*-interaction = 0.604 | *P*-interaction = 0.448 | *P*-interaction = 0.439 | *P*-interaction = 0.409 |
| **BMI <18.5 kg/m^2^** | **Tertile 1** | 35 | 7 | 110.0 | 1 (ref.) | 1 (ref.) | 1 (ref.) | 1 (ref.) |
|  | **Tertile 2** | 55 | 7 | 67.8 | 0.61 (0.21-1.73) | 0.57 (0.20-1.64) | 0.57 (0.20-1.63) | 0.61 (0.21-1.76) |
|  | **Tertile 3** | 20 | 2 | 47.1 | 0.42 (0.09-2.02) | 0.41 (0.09-1.99) | 0.43 (0.09-2.09) | 0.45 (0.09-2.15) |
| **BMI ≥ 18.5 and <25 kg/m^2^** | **Tertile 1** | 586 | 101 | 91.1 | 1 (ref.) | 1 (ref.) | 1 (ref.) | 1 (ref.) |
|  | **Tertile 2** | 888 | 157 | 90.3 | 0.99 (0.77-1.27) | 1.01 (0.79-1.30) | 1.05 (0.82-1.35) | 1.08 (0.84-1.39) |
|  | **Tertile 3** | 569 | 92 | 77.9 | 0.86 (0.65-1.14) | 0.90 (0.68-1.20) | 0.96 (0.72-1.27) | 1.01 (0.76-1.35) |
| **BMI ≥ 25 kg/m^2^** | **Tertile 1** | 345 | 57 | 84.7 | 1 (ref.) | 1 (ref.) | 1 (ref.) | 1 (ref.) |
|  | **Tertile 2** | 726 | 106 | 75.0 | 0.89 (0.64-1.22) | 0.92 (0.67-1.27) | 0.94 (0.68-1.30) | 0.97 (0.70-1.34) |
|  | **Tertile 3** | 729 | 96 | 62.6 | 0.74 (0.53-1.03) | 0.82 (0.59-1.14) | 0.87 (0.62-1.21) | 0.91 (0.65-1.27) |
|  |  |  |  |  | *P*-interaction = 0.832 | *P*-interaction = 0.812 | *P*-interaction = 0.776 | *P*-interaction = 0.791 |
| **SBP < 123 mmHg (median)** | **Tertile 1** | 555 | 103 | 97.0 | 1 (ref.) | 1 (ref.) | 1 (ref.) | 1 (ref.) |
|  | **Tertile 2** | 834 | 148 | 90.9 | 0.94 (0.73-1.21) | 0.98 (0.76-1.26) | 0.99 (0.77-1.27) | 1.03 (0.80-1.33) |
|  | **Tertile 3** | 546 | 113 | 98.8 | 1.02 (0.78-1.34) | 1.12 (0.86-1.47) | 1.13 (0.86-1.48) | 1.20 (0.91-1.57) |
| **SBP ≥ 123 mmHg (median)** | **Tertile 1** | 411 | 62 | 79.2 | 1 (ref.) | 1 (ref.) | 1 (ref.) | 1 (ref.) |
|  | **Tertile 2** | 835 | 122 | 75.0 | 0.95 (0.70-1.28) | 0.97 (0.71-1.31) | 0.97 (0.71-1.32) | 0.98 (0.72-1.33) |
|  | **Tertile 3** | 772 | 77 | 47.8 | 0.60 (0.43-0.84) | 0.67 (0.48-0.94) | 0.67 (0.48-0.94) | 0.70 (0.50-0.98) |
|  |  |  |  |  | *P*-interaction = 0.011 | *P*-interaction = 0.015 | *P*-interaction = 0.016 | *P*-interaction = 0.017 |
| **eGFR < 60 mL/min/1.73m^2^** | **Tertile 1** | 701 | 103 | 77.3 | 1 (ref.) | 1 (ref.) | 1 (ref.) | 1 (ref.) |
|  | **Tertile 2** | 1297 | 194 | 75.7 | 0.98 (0.77-1.25) | 1.00 (0.79-1.28) | 1.03 (0.82-1.32) | 1.05 (0.83-1.34) |
|  | **Tertile 3** | 1059 | 142 | 62.8 | 0.81 (0.63-1.05) | 0.88 (0.68-1.14) | 0.94 (0.73-1.22) | 0.97 (0.75-1.27) |
| **eGFR ≥ 60 mL/min/1.73m^2^** | **Tertile 1** | 265 | 62 | 121.2 | 1 (ref.) | 1 (ref.) | 1 (ref.) | 1 (ref.) |
|  | **Tertile 2** | 372 | 76 | 109.8 | 0.90 (0.65-1.27) | 0.91 (0.65-1.28) | 0.94 (0.68-1.32) | 0.97 (0.70-1.37) |
|  | **Tertile 3** | 259 | 48 | 96.9 | 0.80 (0.55-1.17) | 0.82 (0.57-1.20) | 0.87 (0.60-1.28) | 0.91 (0.63-1.34) |
|  |  |  |  |  | *P*-interaction = 0.919 | *P*-interaction = 0.900 | *P*-interaction = 0.903 | *P*-interaction = 0.932 |
| **Chronic HF** | **Tertile 1** | 473 | 98 | 107.4 | 1 (ref.) | 1 (ref.) | 1 (ref.) | 1 (ref.) |
|  | **Tertile 2** | 764 | 150 | 99.7 | 0.93 (0.72-1.20) | 0.96 (0.75-1.25) | 0.98 (0.77-1.28) | 0.99 (0.77-1.29) |
|  | **Tertile 3** | 621 | 112 | 84.5 | 0.79 (0.60-1.04) | 0.85 (0.65-1.12) | 0.89 (0.68-1.17) | 0.92 (0.70-1.22) |
| **De novo HF** | **Tertile 1** | 493 | 67 | 71.9 | 1 (ref.) | 1 (ref.) | 1 (ref.) | 1 (ref.) |
|  | **Tertile 2** | 905 | 120 | 68.5 | 0.95 (0.71-1.28) | 0.96 (0.72-1.31) | 1.00 (0.74-1.35) | 1.05 (0.78-1.43) |
|  | **Tertile 3** | 697 | 78 | 54.5 | 0.76 (0.55-1.06) | 0.83 (0.6-1.160) | 0.89 (0.64-1.25) | 0.95 (0.68-1.33) |
|  |  |  |  |  | *P*-interaction = 0.953 | *P*-interaction = 0.995 | *P*-interaction = 0.997 | *P*-interaction = 0.954 |
| **Diabetes (-)** | **Tertile 1** | 628 | 106 | 88.3 | 1 (ref.) | 1 (ref.) | 1 (ref.) | 1 (ref.) |
|  | **Tertile 2** | 1059 | 157 | 75.7 | 0.86 (0.67-1.10) | 0.89 (0.70-1.14) | 0.92 (0.72-1.18) | 0.94 (0.74-1.21) |
|  | **Tertile 3** | 897 | 122 | 64.5 | 0.73 (0.56-0.95) | 0.81 (0.62-1.06) | 0.87 (0.67-1.14) | 0.90 (0.69-1.18) |
| **Diabetes (+)** | **Tertile 1** | 338 | 59 | 91.7 | 1 (ref.) | 1 (ref.) | 1 (ref.) | 1 (ref.) |
|  | **Tertile 2** | 610 | 113 | 95.6 | 1.05 (0.77-1.44) | 1.06 (0.77-1.45) | 1.10 (0.80-1.51) | 1.16 (0.84-1.59) |
|  | **Tertile 3** | 421 | 68 | 78.8 | 0.86 (0.61-1.22) | 0.91 (0.64-1.28) | 0.97 (0.69-1.38) | 1.04 (0.73-1.48) |
|  |  |  |  |  | *P*-interaction = 0.604 | *P*-interaction = 0.448 | *P*-interaction = 0.439 | *P*-interaction = 0.409 |
| **IHD (-)** | **Tertile 1** | 266 | 45 | 87.8 | 1 (ref.) | 1 (ref.) | 1 (ref.) | 1 (ref.) |
|  | **Tertile 2** | 546 | 83 | 76.6 | 0.87 (0.61-1.25) | 0.90 (0.63-1.30) | 0.93 (0.65-1.34) | 0.93 (0.65-1.34) |
|  | **Tertile 3** | 449 | 62 | 64.8 | 0.74 (0.50-1.09) | 0.82 (0.56-1.21) | 0.88 (0.60-1.30) | 0.88 (0.60-1.30) |
| **IHD (+)** | **Tertile 1** | 700 | 120 | 90.1 | 1 (ref.) | 1 (ref.) | 1 (ref.) | 1 (ref.) |
|  | **Tertile 2** | 1123 | 187 | 86.1 | 0.96 (0.76-1.20) | 0.98 (0.78-1.23) | 1.02 (0.81-1.28) | 1.06 (0.84-1.33) |
|  | **Tertile 3** | 869 | 128 | 71.1 | 0.79 (0.62-1.02) | 0.85 (0.66-1.10) | 0.91 (0.71-1.18) | 0.98 (0.75-1.26) |
|  |  |  |  |  | *P*-interaction = 0.911 | *P*-interaction = 0.931 | *P*-interaction = 0.915 | *P*-interaction = 0.841 |
| **AF (-)** | **Tertile 1** | 610 | 94 | 80.5 | 1 (ref.) | 1 (ref.) | 1 (ref.) | 1 (ref.) |
|  | **Tertile 2** | 1080 | 172 | 81.5 | 1.01 (0.79-1.30) | 1.04 (0.81-1.34) | 1.08 (0.84-1.39) | 1.10 (0.86-1.42) |
|  | **Tertile 3** | 940 | 126 | 63.5 | 0.79 (0.61-1.03) | 0.86 (0.66-1.13) | 0.91 (0.70-1.21) | 0.95 (0.73-1.26) |
| **AF (+)** | **Tertile 1** | 356 | 71 | 105.0 | 1 (ref.) | 1 (ref.) | 1 (ref.) | 1 (ref.) |
|  | **Tertile 2** | 589 | 98 | 85.6 | 0.81 (0.60-1.11) | 0.83 (0.61-1.13) | 0.86 (0.64-1.18) | 0.89 (0.66-1.22) |
|  | **Tertile 3** | 378 | 64 | 82.9 | 0.79 (0.56-1.11) | 0.86 (0.61-1.21) | 0.92 (0.65-1.30) | 0.95 (0.68-1.35) |
|  |  |  |  |  | *P*-interaction = 0.441 | *P*-interaction = 0.403 | *P*-interaction = 0.416 | *P*-interaction = 0.458 |

The mean daily doses of sacubitril/valsartan were: tertile 1, <24/26 mg twice daily (<100 mg/day); tertile 2, 24/26–83.8/89 mg twice daily (100–172.8 mg/day); and tertile 3, >83.8/89 mg twice daily (>172.8 mg/day).

Model 1 adjusted for age and sex. Model 2 adjusted for age, sex, systolic blood pressure, and body mass index. Finally, model 3 adjusted for all the variables in model 2 as well as comorbidities of hypertension, diabetes, dyslipidaemia, ischemic stroke, intracerebral haemorrhage, atrial fibrillation, myocardial infarction, ischemic heart disease, peripheral artery disease, chronic kidney disease, chronic obstructive pulmonary disease, liver cirrhosis, and cancer.

Abbreviations: BMI, body mass index; CKD, chronic kidney disease; IHD, ischemic heart disease; IR, incidence rate; HF, heart failure; PY, person-year; SBP, systolic blood pressure

**Supplemental Table 5. The risk of all-cause mortality according to various subgroups in multivariable Cox regression analyses.**

|  |  | **N** | **Death** | **IR (per 1,000 PY)** | **Unadjusted model** | **Model 1** | **Model 2** | **Model 3** |
| --- | --- | --- | --- | --- | --- | --- | --- | --- |
| **Age < 40** | Tertile 1 | 19 | 1 | 24.7 | 1 (ref.) | 1 (ref.) | 1 (ref.) | 1 (ref.) |
|  | Tertile 2 | 57 | 2 | 16.9 | 0.70 (0.06-7.71) | 0.65 (0.06-7.18) | 0.67 (0.06-7.35) | 0.74 (0.07-8.21) |
|  | Tertile 3 | 108 | 3 | 12.8 | 0.52 (0.05-5.00) | 0.50 (0.05-4.85) | 0.57 (0.06-5.52) | 0.56 (0.06-5.44) |
| **Age 40-64** | Tertile 1 | 400 | 30 | 35.1 | 1 (ref.) | 1 (ref.) | 1 (ref.) | 1 (ref.) |
|  | Tertile 2 | 775 | 32 | 19.3 | 0.55 (0.33-0.90) | 0.56 (0.34-0.92) | 0.57 (0.35-0.94) | 0.62 (0.37-1.02) |
|  | Tertile 3 | 707 | 19 | 11.7 | 0.32 (0.18-0.57) | 0.35 (0.20-0.62) | 0.36 (0.20-0.64) | 0.39 (0.22-0.70) |
| **Age ≥ 65** | Tertile 1 | 547 | 65 | 58.3 | 1 (ref.) | 1 (ref.) | 1 (ref.) | 1 (ref.) |
|  | Tertile 2 | 837 | 77 | 43.4 | 0.73 (0.52-1.02) | 0.75 (0.54-1.04) | 0.75 (0.54-1.04) | 0.79 (0.56-1.10) |
|  | Tertile 3 | 503 | 48 | 43.4 | 0.72 (0.49-1.04) | 0.72 (0.49-1.04) | 0.73 (0.50-1.06) | 0.79 (0.54-1.15) |
|  |  |  |  |  | *P*-interaction = 0.264 | *P*-interaction = 0.369 | *P*-interaction = 0.401 | *P*-interaction = 0.410 |
| **Male** | Tertile 1 | 682 | 74 | 52.4 | 1 (ref.) | 1 (ref.) | 1 (ref.) | 1 (ref.) |
|  | Tertile 2 | 1179 | 84 | 33.6 | 0.63 (0.46-0.86) | 0.69 (0.51-0.95) | 0.70 (0.51-0.96) | 0.74 (0.54-1.02) |
|  | Tertile 3 | 1023 | 52 | 22.6 | 0.41 (0.29-0.59) | 0.52 (0.37-0.75) | 0.54 (0.37-0.77) | 0.58 (0.40-0.84) |
| **Female** | Tertile 1 | 284 | 22 | 36.7 | 1 (ref.) | 1 (ref.) | 1 (ref.) | 1 (ref.) |
|  | Tertile 2 | 490 | 27 | 25.6 | 0.70 (0.40-1.22) | 0.69 (0.39-1.21) | 0.68 (0.39-1.19) | 0.70 (0.40-1.23) |
|  | Tertile 3 | 295 | 18 | 27.2 | 0.72 (0.39-1.34) | 0.80 (0.43-1.48) | 0.79 (0.42-1.47) | 0.82 (0.44-1.54) |
|  |  |  |  |  | *P*-interaction = 0.278 | *P*-interaction = 0.421 | *P*-interaction = 0.459 | *P*-interaction = 0.482 |
| **BMI <18.5 kg/m2** | Tertile 1 | 35 | 3 | 42.0 | 1 (ref.) | 1 (ref.) | 1 (ref.) | 1 (ref.) |
|  | Tertile 2 | 55 | 7 | 62.6 | 1.49 (0.39-5.76) | 1.52 (0.39-5.89) | 1.55 (0.40-6.03) | 1.59 (0.41-6.23) |
|  | Tertile 3 | 20 | 1 | 22.2 | 0.51 (0.05-4.86) | 0.53 (0.06-5.10) | 0.54 (0.06-5.16) | 0.58 (0.06-5.62) |
| **BMI ≥ 18.5 and <25 kg/m2** | Tertile 1 | 586 | 64 | 53.0 | 1 (ref.) | 1 (ref.) | 1 (ref.) | 1 (ref.) |
|  | Tertile 2 | 888 | 60 | 31.3 | 0.58 (0.41-0.82) | 0.60 (0.42-0.86) | 0.60 (0.42-0.85) | 0.65 (0.45-0.92) |
|  | Tertile 3 | 569 | 44 | 34.6 | 0.62 (0.42-0.91) | 0.70 (0.48-1.03) | 0.70 (0.47-1.03) | 0.77 (0.52-1.14) |
| **BMI ≥ 25 kg/m2** | Tertile 1 | 345 | 29 | 39.5 | 1 (ref.) | 1 (ref.) | 1 (ref.) | 1 (ref.) |
|  | Tertile 2 | 726 | 44 | 28.8 | 0.73 (0.46-1.17) | 0.79 (0.49-1.26) | 0.78 (0.49-1.25) | 0.80 (0.50-1.28) |
|  | Tertile 3 | 729 | 25 | 15.2 | 0.37 (0.22-0.64) | 0.45 (0.26-0.77) | 0.45 (0.26-0.77) | 0.46 (0.27-0.80) |
|  |  |  |  |  | *P*-interaction = 0.124 | *P*-interaction = 0.155 | *P*-interaction = 0.154 | *P*-interaction = 0.143 |
| **SBP < 123 mmHg (median)** | Tertile 1 | 555 | 58 | 49.8 | 1 (ref.) | 1 (ref.) | 1 (ref.) | 1 (ref.) |
|  | Tertile 2 | 834 | 50 | 27.7 | 0.55 (0.38-0.80) | 0.60 (0.41-0.87) | 0.59 (0.41-0.87) | 0.64 (0.44-0.94) |
|  | Tertile 3 | 546 | 29 | 22.9 | 0.44 (0.28-0.68) | 0.53 (0.34-0.82) | 0.54 (0.34-0.84) | 0.58 (0.37-0.91) |
| **SBP ≥ 123 mmHg (median)** | Tertile 1 | 411 | 38 | 44.8 | 1 (ref.) | 1 (ref.) | 1 (ref.) | 1 (ref.) |
|  | Tertile 2 | 835 | 61 | 34.8 | 0.77 (0.52-1.16) | 0.82 (0.55-1.23) | 0.83 (0.55-1.25) | 0.86 (0.57-1.29) |
|  | Tertile 3 | 772 | 41 | 24.2 | 0.52 (0.34-0.81) | 0.65 (0.42-1.01) | 0.67 (0.43-1.04) | 0.72 (0.46-1.13) |
|  |  |  |  |  | *P*-interaction = 0.480 | *P*-interaction = 0.522 | *P*-interaction = 0.495 | *P*-interaction = 0.578 |
| **eGFR < 60 mL/min/1.73m^2^** | **Tertile 1** | 701 | 63 | 43.6 | 1 (ref.) | 1 (ref.) | 1 (ref.) | 1 (ref.) |
|  | **Tertile 2** | 1297 | 66 | 23.8 | 0.53 (0.38-0.76) | 0.57 (0.41-0.81) | 0.57 (0.41-0.82) | 0.61 (0.43-0.86) |
|  | **Tertile 3** | 1059 | 38 | 15.8 | 0.34 (0.23-0.52) | 0.41 (0.28-0.62) | 0.42 (0.28-0.64) | 0.45 (0.30-0.68) |
| **eGFR ≥ 60 mL/min/1.73m^2^** | **Tertile 1** | 265 | 33 | 58.3 | 1 (ref.) | 1 (ref.) | 1 (ref.) | 1 (ref.) |
|  | **Tertile 2** | 372 | 45 | 57.4 | 0.99 (0.63-1.56) | 1.00 (0.64-1.57) | 0.98 (0.63-1.55) | 0.99 (0.63-1.57) |
|  | **Tertile 3** | 259 | 32 | 57.8 | 0.98 (0.61-1.61) | 1.00 (0.62-1.64) | 1.02 (0.63-1.66) | 1.06 (0.65-1.75) |
|  |  |  |  |  | *P*-interaction = 0.004 | *P*-interaction = 0.019 | *P*-interaction = 0.022 | *P*-interaction = 0.026 |
| **Chronic HF** | **Tertile 1** | 473 | 57 | 56.1 | 1 (ref.) | 1 (ref.) | 1 (ref.) | 1 (ref.) |
|  | **Tertile 2** | 764 | 61 | 36.4 | 0.64 (0.45-0.92) | 0.69 (0.49-1.00) | 0.70 (0.49-1.01) | 0.70 (0.49-1.02) |
|  | **Tertile 3** | 621 | 39 | 27.0 | 0.46 (0.31-0.70) | 0.54 (0.36-0.82) | 0.54 (0.36-0.83) | 0.56 (0.37-0.86) |
| **De novo HF** | **Tertile 1** | 493 | 39 | 39.2 | 1 (ref.) | 1 (ref.) | 1 (ref.) | 1 (ref.) |
|  | **Tertile 2** | 905 | 50 | 26.6 | 0.67 (0.44-1.02) | 0.70 (0.46-1.07) | 0.70 (0.46-1.07) | 0.77 (0.51-1.19) |
|  | **Tertile 3** | 697 | 31 | 20.4 | 0.50 (0.31-0.81) | 0.62 (0.39-1.01) | 0.63 (0.40-1.03) | 0.71 (0.44-1.17) |
|  |  |  |  |  | *P*-interaction = 0.965 | *P*-interaction = 0.872 | *P*-interaction = 0.864 | *P*-interaction = 0.759 |
| **Diabetes (-)** | Tertile 1 | 628 | 57 | 43.5 | 1 (ref.) | 1 (ref.) | 1 (ref.) | 1 (ref.) |
|  | Tertile 2 | 1059 | 71 | 31.7 | 0.72 (0.51-1.02) | 0.79 (0.56-1.12) | 0.79 (0.56-1.13) | 0.83 (0.58-1.171) |
|  | Tertile 3 | 897 | 42 | 20.7 | 0.46 (0.31-0.68) | 0.58 (0.39-0.87) | 0.60 (0.40-0.90) | 0.63 (0.42-0.95) |
| **Diabetes (+)** | Tertile 1 | 338 | 39 | 55.5 | 1 (ref.) | 1 (ref.) | 1 (ref.) | 1 (ref.) |
|  | Tertile 2 | 610 | 40 | 30.5 | 0.54 (0.35-0.84) | 0.55 (0.36-0.86) | 0.55 (0.35-0.86) | 0.60 (0.39-0.94) |
|  | Tertile 3 | 421 | 28 | 30.0 | 0.52 (0.32-0.85) | 0.56 (0.35-0.92) | 0.57 (0.35-0.93) | 0.63 (0.39-1.04) |
|  |  |  |  |  | P-interaction = 0.364 | *P*-interaction = 0.400 | *P*-interaction = 0.391 | *P*-interaction = 0.470 |
| **IHD (-)** | Tertile 1 | 266 | 20 | 35.5 | 1 (ref.) | 1 (ref.) | 1 (ref.) | 1 (ref.) |
|  | Tertile 2 | 546 | 26 | 22.2 | 0.62 (0.35-1.11) | 0.67 (0.37-1.19) | 0.67 (0.37-1.20) | 0.67 (0.38-1.21) |
|  | Tertile 3 | 449 | 17 | 16.8 | 0.46 (0.24-0.87) | 0.57 (0.30-1.09) | 0.58 (0.30-1.11) | 0.57 (0.30-1.09) |
| **IHD (+)** | Tertile 1 | 700 | 76 | 52.5 | 1 (ref.) | 1 (ref.) | 1 (ref.) | 1 (ref.) |
|  | Tertile 2 | 1123 | 85 | 35.7 | 0.67 (0.49-0.92) | 0.71 (0.52-0.97) | 0.71 (0.52-0.97) | 0.75 (0.55-1.02) |
|  | Tertile 3 | 869 | 53 | 27.2 | 0.50 (0.35-0.71) | 0.59 (0.41-0.83) | 0.60 (0.42-0.86) | 0.65 (0.45-0.94) |
|  |  |  |  |  | *P*-interaction = 0.960 | *P*-interaction = 0.983 | *P*-interaction = 0.983 | *P*-interaction = 0.924 |
| **AF (-)** | **Tertile 1** | 610 | 59 | 46.7 | 1 (ref.) | 1 (ref.) | 1 (ref.) | 1 (ref.) |
|  | **Tertile 2** | 1080 | 79 | 34.4 | 0.72 (0.52-1.02) | 0.79 (0.56-1.11) | 0.79 (0.57-1.12) | 0.81 (0.58-1.15) |
|  | **Tertile 3** | 940 | 46 | 21.7 | 0.44 (0.30-0.65) | 0.53 (0.36-0.78) | 0.54 (0.37-0.81) | 0.57 (0.39-0.85) |
| **AF (+)** | **Tertile 1** | 356 | 37 | 49.4 | 1 (ref.) | 1 (ref.) | 1 (ref.) | 1 (ref.) |
|  | **Tertile 2** | 589 | 32 | 25.4 | 0.51 (0.32-0.82) | 0.52 (0.33-0.85) | 0.52 (0.33-0.84) | 0.58 (0.36-0.94) |
|  | **Tertile 3** | 378 | 24 | 28.6 | 0.56 (0.34-0.94) | 0.67 (0.40-1.13) | 0.68 (0.41-1.15) | 0.79 (0.47-1.35) |
|  |  |  |  |  | *P*-interaction = 0.181 | *P*-interaction = 0.127 | *P*-interaction = 0.127 | *P*-interaction = 0.125 |

The mean daily doses of sacubitril/valsartan were: tertile 1, <24/26 mg twice daily (<100 mg/day); tertile 2, 24/26–83.8/89 mg twice daily (100–172.8 mg/day); and tertile 3, >83.8/89 mg twice daily (>172.8 mg/day).

Model 1 adjusted for age and sex. Model 2 adjusted for age, sex, systolic blood pressure, and body mass index. Finally, model 3 adjusted for all the variables in model 2 as well as comorbidities of hypertension, diabetes, dyslipidaemia, ischemic stroke, intracerebral haemorrhage, atrial fibrillation, myocardial infarction, ischemic heart disease, peripheral artery disease, chronic kidney disease, chronic obstructive pulmonary disease, liver cirrhosis, and cancer.

Abbreviations: BMI, body mass index; CKD, chronic kidney disease; IHD, ischemic heart disease; IR, incidence rate; HF, heart failure; PY, person-year; SBP, systolic blood pressure
